# The role of non-state actors in combating COVID-19 spread in Northern Jordan

**DOI:** 10.1016/j.amsu.2020.11.005

**Published:** 2020-11-09

**Authors:** Adi H. Khassawneh, Nasr Alrabadi, Abdel-Hameed Al-Mistarehi, Nail Obeidat, Khalid A. Kheirallah

**Affiliations:** a Department of Public Health and Family Medicine, Faculty of Medicine, Jordan University of Science and Technology, Irbid, Jordan; b Department of Pharmacology, Faculty of Medicine, Jordan University of Science and Technology, Irbid, Jordan; c Department of Obstetrics and Gynecology, Faculty of Medicine, Jordan University of Science and Technology, Irbid, Jordan

The coronavirus disease 2019 (COVID-19) pandemic is a global problem where all countries should react appropriately and participate effectively to deal with it. In Jordan, a small country with a population of 12.2 million in the Middle East, after the first case with COVID-19 infection was reported on March 2nd, 2020, the government initiated a two-week quarantine for arrivals from high-risk countries. On March 15th, as a response to the increased number of reported new cases, the government further adopted a series of strict Non-Pharmaceutical Intervention (NPI) measures to mitigate the spread of COVID-19. These included extensive contact tracing and quarantining of contacts within their households for two weeks if tested negative. Besides, all laboratory-confirmed COVID-19 cases, even if asymptomatic, were forced to mandatory quarantine within designated hospitals [1,2].

On March 17th, 2020, the number of reported confirmed COVID-19 cases reached 40. This called on the Jordanian government to take several additional NPI measures including activating the National Defence Law. Accordingly, a strict countrywide lockdown and nationwide curfew were imposed. This curfew prohibited people’s movement, implemented a stay-at-home policy, stopped public, social, and religious events, closed all shops, and suspended both governmental and non-governmental institutions except for vital sectors [1,3]. During the first couple of days of the curfew, the residents were prevented from leaving their homes except for only health emergencies [3]. They were then allowed five specific days per week to move around and walk in their geographic boundaries between 10 a.m. and 6 p.m. [3]. People who were found in violation of the curfew order would be immediately imprisoned for up to a year.

Although these strict state NPI measures including lockdown and curfew were successful in combating the spread of the infection [1,3] (Fig. 1), they had significant impacts on the population including difficulty in reaching their daily needs, the inability of individuals with chronic medical illnesses to reach their medications’ supplies, and obstacle dealing with health emergencies as well as the psychological impact of the COVID-19 outbreak and its related lockdown measures and the home quarantine of unknown duration on the general population. During the lockdown, the residents went through a range of psychological and emotional reactions, loss of income, fear, and uncertainty being one of the infected cases. A recent study from Italy assessed the mental health effects of three to 4 weeks lockdown measures in the general Italian population and reported high rates of post-traumatic stress symptoms, depression, anxiety symptoms, insomnia, perceived stress, and adjustment disorder symptoms [5].

**Fig. 1:**
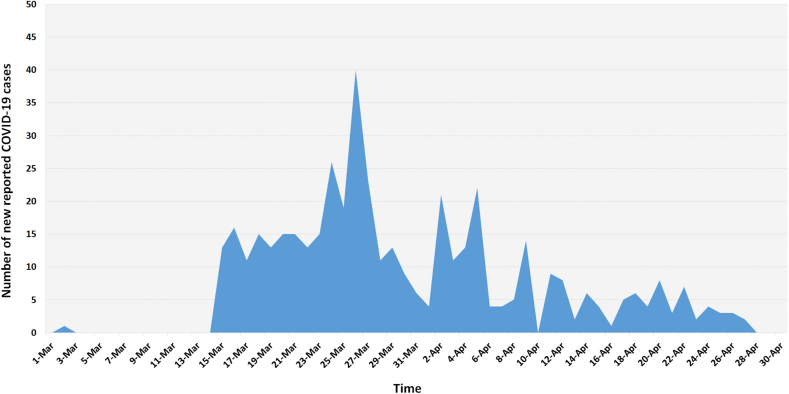
The number of daily new confirmed COVID-19 cases in Jordan, regardless of their nationalities [Source: Wikipedia [4]].

The country's hardest-hit part was the northern part of Jordan, particularly Irbid city which is Jordan's second-most populous. Therefore, in response to the localized spread of the COVID-19 epidemic in Northern Jordan and in order to relieve some of the workloads on healthcare workers, and to mitigate the curfew effects on people; a medical Initiative was launched to provide supportive healthcare services and medical consultations, and to deliver medications to patients with chronic medical conditions. This Initiative, called “Himmat Wattan”, was launched by the Jordanian Medical Association (JMA)-Irbid Branch and played a significant role in combating the spread of COVID-19 and in alleviating the impact of strict state measures on the general health of the public.

This report presents the activities conducted by “Himmat Wattan” in Northern Jordan. It highlights the role of Non-State Actors in activating a localized response that supported the healthcare workers’ medical mission and strengthened the response in fighting COVID-19 infection when a curfew has paralyzed essential medical services.

During the period from 19th to 25th, March 2020, the Initiative began with JMA members by collecting monetary donations and purchasing personal protective equipment (PPE) in high demand which included: N95 Masks, Gloves, Gowns, Face shields, Eye goggles, Sterilizers, etc. then distributing them to frontline healthcare workers. However, reaching these items to buy was difficult as most pharmacies and shops complained of a shortage in these items. Therefore, to solve this problem we contacted the industries to get the required PPE. Further, training sessions on the proper selection and use of PPE were provided to frontline healthcare workers and allowed participants to practice the correct way to don and remove PPE. These training sessions improved personnel safety in the healthcare environment and increased awareness among frontline healthcare workers in how to protect themselves and their patients against infectious materials. As gloves are the most common type of PPE used in healthcare settings, we shed light on the importance of changing them after use on each patient, or If torn or when heavily soiled even during use on the same patient, and how to Limit opportunities for touch contamination. These sessions made a noticeable improvement in the practice of healthcare workers and kept them up-to-date with COVID-19 preventive protocols.

Since the 26th of March, the Initiative started to engage members from outside JMA (mainly dentists, nurses, and medical students) and conducted the first broad meeting to draw a road map to adopt new activities and fine-tune existing ones. These activities included 1) supported the civil defense emergency services by providing trained physicians within ambulances to deal with suspected or confirmed COVID-19 cases; 2) established communication centers to receive 911-calls in order to provide tailored medical consultation and advice, triage patients' complaints into an emergency or non-emergency, and correctly dispatch and guide ambulances to patients with emergency needs. This approach led to a significant decrease in the number of hospital transfers from 600 to 700/day before the Initiative to 80–90/day during the Initiative. As well, Initiative 3) established a media center to orient the public about COVID-19 mitigation and preventive measures and to identify rumors and warn against the wrong infodemic. The Initiative also 4) established what so-called “movement team” to support contact tracing by evaluating the different geographical areas’ infectious status using a time-sensitive risk assessment matrix and giving movement orders to field teams to investigate suspected cases and potential contacts. Further, sub-committees were established from the Initiative members and the public to guide fundraising activities and evaluate the shortage and the supply needs for emergency rooms of hospitals and centers dealing with COVID-19 patients.

Similarly, the steering committee of the Initiative signed a Memorandum of Agreement with major specialized hospitals in North of Jordan to distribute filled prescriptions not only to chronic disease patients but also to oncology patients and transplant patients. The daily number of prescriptions ranged between three and four thousand. This Agreement is designated to be still valid during medical emergencies and establishes clear guidelines, responsibilities, and roles for non-state actors.

Throughout the Initiative, the number of participants increased from around 30 JMA members to more than 500 members. Volunteers were directly participating in almost all activities of the Initiative but mainly field investigation and contact tracing. As part of the continuous efforts in dealing with the COVID-19 pandemic, the medical teams focused more on community awareness and efforts towards controlling the pandemic. On April 19th, a massive safety campaign, “Salamatuna Fe Tba'adouna” (our safety in our distancing), was established, where a big group of volunteers launched a community awareness campaign that included many trained and professional teams of medical students and physicians. They held awareness lectures within public places such as shopping malls and grocery stores, raised awareness about the importance of social distancing, distributed flyers, posters, and sterilizers and installed safety belts in crowded places. This was even improved on July 10th, when an agreement has been signed with the Forum of Artists to allocate a substantial educational wall-murals to be implemented starting from the beginning of August. This aimed at consolidating the concepts of social distancing and awareness of COVID-19 and honor everyone who had a role in the fight against the pandemic.

However, we faced several challenges during the conduction of this Initiative. First, the relatively small number of JMA members to participate, and we solved this issue by inviting members from outside JMA (mainly dentists, nurses, and medical students) to be part of our project. Second, the financial resources were limited and there was a shortage of PPE in the markets; we overcame this by collecting monetary donations and going to the industries to purchase the required PPE. Third, we overcame the need for vehicles to transport the medications and PPE to the target populations by depending on the volunteers’ private cars. Forth, the lack of emergency management specialists to run the Initiative was evident as decisions for proper implementation of activities were not efficient, sometimes, and corrective action was necessary in times where a limitation presented itself. Finally, the need to implement mental health counseling and services to the general population during the lockdown was not met as such services are considered a taboo during normal circumstances. Lack of professionals to implement such services also played a role in designing such interventions. Without proper guidance, this service may not work and fire back at the activities of the Initiative.

Overall, volunteers and non-state actors play an essential role in mitigating the effects of the COVID-19 outbreak and its related lockdown measures on the general population and helping the hospitals, civil defense emergency services, and contact tracing teams run smoothly and relieving workloads on healthcare workers. This Initiative successfully reduced the number of hospital transfers from 911-calls by more than 85% and ensured that medications for chronic diseases are timely disseminated. It also played a role in fighting rumors about COVID-19 and fought against inaccurate information about its treatment and preventive measures. Overall, the Initiative increased the understanding, and value, of the role non-state actors can play during public health emergencies and the real need to volunteer to meet the general population’s needs when critical needs arise.

This report also highlighted the leading roles of the Initiative in dealing with COVID-19 outbreaks, still, it provided a guideline for the future establishment of training materials for emergency preparedness and response. The efforts presented in this report are the first evidence of non-state actors’ role in dealing with COVID-19 and establishes continued efforts towards facing the COVID-19 pandemic and lessons learned for better accommodation with emergencies in the future.

## Provenance and peer review

Not commissioned, externally peer-reviewed.

## Ethical approval

It is a short communication about the role of Non-State Actors in Combating COVID-19 Spread in Northern Jordan, not require institutional review board or consent.

## Sources of funding

No funding was received for this article.

## Author contribution

All authors contributed significantly and in agreement with the content of the article. All authors were involved in project design, data collection, data interpretation, and writing the manuscript. All authors presented substantial contributions to the short communication and participated in correction and final approval of the version to be submitted.

## Registration of research studies

Not require registration as it is a short communication that does not involve human participants.

## Guarantor

Adi Khassawneh. Email: ohkhasawneh@just.edu.jo.

## Declaration of competing interest

The authors declare that they have no competing interests.
